# The Roles of Community Health Nurses’ in Covid-19 Management in Indonesia: A Qualitative Study

**DOI:** 10.30476/IJCBNM.2021.90884.1739

**Published:** 2022-04

**Authors:** M. Agung Akbar, Neti Juniarti, Ahmad Yamin

**Affiliations:** 1 Department of Community Health Nursing, Academy of Nursing Al-Maarif, Baturaja, Indonesia; 2 Department of Community Health Nursing, Faculty of Nursing, Universitas Padjadjaran, Bandung, Indonesia

**Keywords:** Community Health Centers, Community Health Nursing, Covid-19

## Abstract

**Background:**

The majority of Covid-19 cases occur at the community level requiring health services to be available at the primary health care level, which also includes Community Health Nursing (CHN) services. It is important to understand various perspectives of the parties involved, effective solutions, and strategies used by nurses in managing Covid-19 in order to be able to provide these services. The purpose of this study was to explore the community health nurses’ (CHNs) roles in the Covid-19 management in Indonesia.

**Methods:**

A qualitative method was used to explore the perspective from nurses and health cadres as participants. Data were collected through in-depth phone interviews with 11 participants from December 2020 to February 2021 in Bandung. Data were analyzed manually using thematic analysis.

**Results:**

Five themes were extracted in this study, namely providing comprehensive services by CHNs; utilizing technology to bridge the information needs; implementing family nursing care; spreading the wings of health cadres by CHNs; and collaborating as the heart of Covid-19 prevention and management.

**Conclusion:**

CHNs should employ health education, empowerment strategies, group processes, and advocacy in adapting to the Covid-19 pandemic situation. The five themes identified in this study can be used by policy makers to develop strategies in optimizing the CHN in Covid-19 pandemic management and the possible challenges of future global pandemics.

## INTRODUCTION

The World Organization’s Emergency Committee has declared coronavirus disease-2019 (Covid-19) as a global health emergency based on the global increase in Covid-19 case notification rates. Due to the global spread of this disease, World Health Organization (WHO) categorizes Covid-19 as a pandemic with a high-risk global scale based on its risk assessment on the disease situation worldwide. ^1^ On 22 June 2021, the Government of the Republic of Indonesia reported 2.018.113 cases with confirmed Covid-19. Totally, 55.291 deaths related to Covid-19 was reported and 1.810.136 patients recovered from the disease. ^2^

This increase in the number of cases is not comparable with the available capacity of the health care system in almost all countries and this situation potentially brings negative consequences for the health workers who have to work amidst the high-risk for infection and limited resources that leads to the inability to provide proper patient management in hospitals. ^3^ The Indonesian Ministry of Health in their study demonstrated that most Covid-19 cases were identified at the community level and required most patients to do self-isolation or home quarantine. ^4^

The fact that most cases are managed at the community level has required the Community Health Center (CHC) to play a role in improving the prevention of the disease transmission and controlling infections at the community level up to the optimum level. Strengthening the CHC capacity is an important part of the overall efforts to achieve the optimum health. ^4 , 5^ One of the important roles of the CHC is providing nursing services. This emphasizes the importance of the Community Health Nurses (CHNs) with the purpose of improving health and reducing health inequality in the community. ^6^

Community Health Nursing (CHN) activities is an integrated part of the CHC activities. CHNs have the duty to provide nursing care to each group in the community with the intention to improve the community’s quality of life in dealing with various problems that cause vulnerability to health problems and health risks. ^6^ However, the needs of today’s society are increasingly complex due to the requirement to adapt to changing times. CHN must be able to apply an integrated and holistic approach in responding to the existing challenges. ^7^

Previous studies explained that nurses’ experiences and understanding of various aspects of this disease have been examined, such as in Covid-19 ward, ^8^ isolation rooms, ^9^ emergency departments, and intensive care units. ^10^ Of course, most of the mentioned studies have been outside CHC. However, the problematic challenges faced by society today require the CHNs, especially in dealing with the challenges of potential public health emergencies. ^11^ During an outbreak, the CHNs need to acquire several competencies that include the skills needed to identify the outbreak. ^12^ Yet, despite the critical role of the CHNs, there was limited evidence about the CHNs’ roles during Covid-19 pandemic. There were also the burnout of the CHN workforce that has made people in the community more vulnerable to both chronic diseases and emerging infectious disease threats. ^13^ Thus, a study is needed to explore the roles of CHNs in Covid-19 management as the evidence to ensure nursing as the core public health workforce in maintaining critical services in communities.

A qualitative study can explore the role of CHNs in Covid-19 pandemic management. This approach can extract a lot of meaningful information and help explain the process to be identified. Qualitative research plays an important role in understanding the different perspectives of the people involved and identifying the effective solutions, and strategies used in an effective way. ^14^ In Indonesia, there are subjective and multiple realities in understanding the meaning of the roles of the CHNs from various stakeholders’ perspectives. From a qualitative perspective, there are multiple realities and truths in understanding the meaning of the nurses’ roles as perceived by different stakeholders. Each stakeholder has had a different experience and holds different views about the management of Covid-19, which should be appreciated and considered as a reality. These multiple realities are constructed based on the social and historical construction of CHN practice in Indonesia. Therefore, the aim of this qualitative study was to explore the roles of CHNs in the Covid-19 management in Indonesia.

## MATERIALS AND METHODS

This was a qualitative exploratory descriptive study conducted from December 2020 to February 2021 in Bandung, Indonesia. The study sites were recommended by the Health Office with reference to the CHC performance assessment results, Covid-19 management at the CHC, and the willingness of the authorities to participate as a study site. As a result, there were 4 study sites selected including the health office, and 3 out of 75 CHC were recommended according to the criteria as a study site. The CHC location was chosen because it is a work area for the nurses in community setting, thus hospital nurses were excluded from this study.

Participants in this study were CHN coordinator as the key informants, CHNs who were in charge of Covid-19 management as the main informants, and the health cadres as supporting informants. In Indonesia, the health cadres are also known as community health volunteers. The characteristics of health cadres are community members who are willing, able, and have time to help organize health service activities voluntarily. The health staff were selected based on the inclusion criteria including at least 1 year experience as a health volunteer, participation in CHN staff training, and involvement in controlling and preventing Covid-19 in the community. The exclusion criterion was unwillingness to participate in the study. 

Each CHC has 5-7 nurses, so the selection of nurses was determined based on the inclusion and exclusion criteria. The participants in this study were recruited using the purposive sampling method; the inclusion criteria were the nurses who had been working in the CHC and were assigned in CHN for a minimum of 1 year; were involved in Covid-19 management in CHC and were able to communicate well and cooperate. The exclusion criteria were unwillingness to participate in the study. The participants were the nurses and health cadres listed at a CHC who met the inclusion and exclusion criteria. In addition, we chose the selected participants at the city health office. In total, eleven subjects participated in this study (Table 1). All participants consented to be involved in the study.

**Table 1:** Participants’ characteristics

Participant Code	Sex	Age (years)	Education Level	Participant Type	Workplace	Length of work (years)
P1	Female	29	Bachelor of Nursing	Nurses	Bandung City Health Office	3.5
P2	Female	40	Bachelor of Nursing	Nurses	Garuda CHC	16
P3	Female	36	Nursing Diploma	Nurses	Kopo CHC	9
P4	Male	30	Bachelor of Nursing	Nurses	Griya Antapani CHC	3
P5	Male	29	Nursing Diploma	Nurses	Kopo CHC	10
P6	Female	42	Nursing Diploma	Nurses	Garuda Public CHC	17
P7	Female	25	Nursing Diploma	Nurses	Kopo CHC	4
P8	Female	41	Nursing Diploma	Nurses	Griya Antapani CHC	10
P9	Female	42	Bachelor of Nursing	Nurses	Griya Antapani CHC	14
P10	Female	45	Senior High School	Cadre	Garuda area	11
P11	Female	46	Junior High School	Cadre	Kopo area	10

Recruitment of the participants was done through the CHN coordinator at each CHC. Data collection method used in this study was In-depth phone interview. ^15^ This phone interview was selected to minimize direct contact between the interviewer and participant during the Covid-19 pandemic, so that virus transmission could be prevented. The schedule for interview was agreed beforehand with the participants according to their convenience. The interview lasted between 20-65 minutes and all interviews were digitally recorded after the participants’ informed consent. After interview with 11 participants, data collection reached saturation and no new categories emerged from the data anymore. 

During the research process, the first author was the interviewer and has previously attended several online qualitative seminars and had been trained in interview techniques by the supervisors. The two supervisors had a Doctor of Philosophy degree and a specialist in community health nursing. Both supervisors focused on the field of CHN and were experts in qualitative research in nursing. All authors checked the processes of interviewing, coding, categorizing, and interpreting the findings. For gathering the data during the interview sessions, an interview guide was provided by reviewing the related literature and consulting with supervisors. 

Data were collected through semi-structured interviews. The interview started with general questions such a “How did CHNs participate in Covid-19 management?”. The interview was then continued to more specific questions such as “How was the involvement of CHNs in health promotion in Covid-19 outbreak?”, “How was the involvement of CHNs in preventing Covid-19 disease?”, “How was the involvement of CHNs in the management of Covid-19 patients?”, and “How was the involvement of CHNs in policy management in Covid-19 outbreak?”

Data were analyzed manually using thematic analysis. ^16 , 17^ This approach provided a rich, detailed, yet complex account of data, and helped to produce a clear and organized final report compared to content analysis. ^16^ This analysis involved six phases of thematic analysis developed by Braun and Clarke, namely familiarization, coding, generating the themes, reviewing the themes, defining and naming the themes, and writing up. ^16^ In the first phase, each interview was transcribed verbatim; we understood the data, took preliminary notes, and generally looked at the data to get familiar with it. The coding phase involved the initial production of codes from the data and focused on specific characteristics of the data. The third phase involved sorting all the potentially relevant coded data extracts into themes. During the fourth phase, researchers reviewed the coded data extracts for each theme and recognized that the theme specified was an accurate representation of the data. In the fifth phase, researchers determined the name of the themes, and the data of each theme were captured. The last phase was writing up of the report. Each phase of data analysis was the contribution of input from all research team after discussion and joint efforts.

To ensure that the result of this study is valid and can be considered as evidence, we established the rigor and trustworthiness of the study based on credibility, transferability, dependability, and confirmability. ^18^ Credibility was achieved by using experiences from various perspectives of the participants. With these approaches, the researchers discussed the findings with participants to ensure the accuracy of data using phones calls. All participants were satisfied with the results. In terms of transferability, the participants with different views were then probed further to understand their views as those views could not be generalized. Strategies to support transferability were performing detailed description of context and analysis process. Dependability indicates that the findings are consistent and reliable. To ensure dependability, researchers clearly defined the study sages and the data were continuously evaluated to ensure accurate data coding. Confirmability was ensured by reflecting information gained on related scientific articles, consulting experts, and confirming with participants. ^19^

This study obtained ethics committee approval from the Research Ethics Committee of Universitas Padjadjaran with the number 1144/UN.6KEP/EC/2020. The authors ensured that all participants were informed about this study and written informed consent was obtained from all the participants. The researchers guaranteed their data confidentiality and ensured them that their information would be published anonymously.

## RESULTS

The results of this study indicated the importance of the involvement of CHNs in managing Covid-19 pandemic through the comprehensive intervention strategy. This study identified fifth themes that emphasized the role of CHN in Covid-19 management in Bandung City, West Java Indonesia. These themes, as well as their sub-themes, are described below (Table 2). 

**Table 2:** Themes and subthemes

Sub Themes	Theme
a. Initial Covid-19 screening for health services	Providing comprehensive services by CHNs
b. Vulnerable group management services	
c. Covid-19 contact tracing in the community	
d. Readiness of nurses to implement Covid-19 vaccination program	
e. Selective home visit	
f. Optimizing services in the CHC building	
a. Utilization of social media	Utilization of technology to bridge the information needs
b. Online nursing monitoring during self-isolation and teleconsultation	
a. Educating families on how to do self-isolation at home	Implementing family nursing care
b. Family nursing care to increase family independence	
a. Helping CHNs to empower health cadres to educate the community regarding Covid-19	Spreading the wings of health cadres by CHNs
b. Helping CHNs to empower health cadres to trace Covid-19 case in the community	
c. Helping CHNs to empower health cadres to control stigma in the community	
a. Collaboration with Non-Government Organization (NGO) to train CHC health workers on 3T (Tracing, Testing, Treatment)	Collaboration as the heart of Covid-19 prevention and management
b. Collaboration with NGO for health education and community based surveillance training for people in the community	
c. Collaboration with the local Covid-19 task force	
d. Optimizing of human resource allocation through collaboration	

### 1. Providing Comprehensive Services by CHNs

This theme describes the providing comprehensive services by CHNs in preventing and managing Covid-19 pandemic at the primary health care facility such as CHC. This first theme was supported by 6 sub- themes: initial Covid-19 screening for health services; vulnerable group management services; Covid-19 contact tracing in the community; readiness of nurses to implement Covid-19 vaccination program; selective home visit; and optimizing services in the CHC building.

#### Initial Covid-19 Screening for Health Services

The participants implemented prevention by screening for Covid-19 before providing services within or outside the CHC building. Participant 6 stated that *“The CHN effort also includes screening for visitors who come to the CHC; then, at the clinic, we prepare forms containing questions on Covid to be answered by the patients”*.

Apart from this, CHN nurses also performed screening when providing services outside the building. This was expressed by Participant 2 who claimed that *“ Indeed, there are mass rapid (test) activities such as in markets, you see. We also do screening (random antigen/antibody examination)” (P2)*.

### Vulnerable Group Management Services

Attention to those with vulnerable group is important because they are at risk of Covid-19 infection. This was reflected in a statement from Participant 6: *“This group is susceptible to infection, so the Chronic Disease Patient Management Program must not be interrupted to keep their condition stable” (P6)*.

The vulnerable group management was performed through activities in the CHC building and periodic activity scheduling as mentioned by Participant 2: *“ Activities are performed in the (CHC) building. But this time, we limit the number of participants, not all, and we make a schedule so that all group members can have their turn to participate in the activities.* Furthermore, all activities were performed while applying the health protocols. This was expressed by Participant 6: *“The program continues, but with health protocol implementation (P6)*.

### Covid-19 Contact Tracing in the Community

The active involvement of the CHNs in contact tracing of Covid-19 cases is crucial in the effort to stop Covid-19 transmission in the community. Participant 6 stated: *“Moreover, we, nurses, are engaged in surveillance” (P6)*. In addition, the tracing was performed according to the area division for each nurse-in-charge for CHN. Participant 8 stated: *“We have five areas under our supervision and the nurse-in-charge for each area does the tracing” (P8)*.

### Readiness of Nurses to Implement Covid-19 Vaccination Program

This sub-theme emerged in the study because the time of data collection coincided with the implementation of the vaccination program in Indonesia. This sub-theme consisted of the participants’ expressions about their self-readiness to implement the vaccination program. Participant 6 stated: *“Regarding the vaccination, yes I agree because the right way to deal with the pandemic is through vaccine, as one of the measures, although it is not the only weapon (P6)*.

Nurses’ readiness to implement the vaccination program was also supported by the fact that they had been trained as vaccinators, as mentioned by Participant 3: *“ We have received distance training (online) about vaccination, yes, at the beginning of the year. The plan is to give vaccination for all residents aged 18 -59 years old in our work area.” (P3)*.

### Selective Home Visit

The online approach cannot substitute all services required by the community. The CHNs also performed selective home visits while applying strict health protocols to the patients’ homes confirmed to be positive for Covid-19 and they underwent self-isolation. This selective visit was performed when the patient’s condition worsened or when the patient was dealing with problems that needed the support from a nurse. This was expressed by Participant 5: *“ In terms of our home visits, they are selective. If the patient’s condition worsens, we will go there” (P5)*.

### Optimizing Services in the CHC Building

In dealing with obstacles outside the CHC building to provide nursing care outside the building, the CHNs attempted to optimize service provision in the CHC building. Participant 7 stated: *“We still provide nursing care, even though it is not 100%; we provide the care in the (CHC) building” (P7)*. In addition, service provision in the CHC was prioritized for poor people that could not be reached by online communication. Participant 4 stated that: *“Actually, for patients who do not have cellphones, phone credit, or the like, like poor families, we insisted that they should be able to visit the CHC if necessary” (P4)*.

Based on the above statement, it was apparent that nurses continued to provide health services by optimizing service provision in the CHC building, especially for the poor that cannot be reached online.

### 2. Utilization of Technology to Bridge Information Needs

This second theme explained that the CHNs could provide online education on Covid-19 to the community. There were two sub-themes found in this theme: utilization of social media and online nursing monitoring during self-isolation and teleconsultation.

#### Utilization of Social Media

Online health education via social media was the main strategy expressed by the majority of participants. This was because social media was widely used by the people in the community. Participant 5 mentioned that: *“ Then, we also educate through social media such as through counseling videos, leaflets. We try to do the program online and not face to face (P5)*.

Health education was provided, especially, through the community’s WhatsApp group as described by Participant 6: *“It can be through active counseling via groups such as WhatsApp”(P6)*. The expressions from several participants above indicated the usefulness of online health education as a strategy for providing Community Service while adapting to the current pandemic situation.

### Online Nursing Monitoring During Self-isolation and Teleconsultation

In an effort to limit direct contact with patients who were tested positive for Covid-19, online contact was used as a strategy used to monitor those who were in self-isolation. Participant 3 stated: *“…and we monitor patients who are Covid-19 positive and are in self-isolation at home. So far, the monitoring that we do is performed using WA (WhatsApp) or by phone (P3)*”

During online monitoring by nurses, anamnesis on the activities performed by the patient/family in self-isolation was conducted. This was reflected in the statement from Participants 7: *“Monitoring is performed online, usually every day, patients fill out the google form to convey whether they are asymptomatic, whether they have complaints today, (to report) fever or symptoms that lead to Covid, that’s what we monitor every day” (P7)*. The online approach to monitoring patients in self-isolation due to Covid-19 in the community is very helpful for implementing patient monitoring by nurses. 

Teleconsultation was used by the CHNs to provide services to the community during the pandemic period, as reflected in the expression from Participant 6: *“we also do counseling for these patients. I do video calls and talk to them during the call” (P6)*. In addition to consultation services related to Covid-19, teleconsultation services provided by the CHNs can also include other public health services. This was expressed by Participant 5 who stated: *“Regarding the pandemic, I propose an innovation for controlling the asthma attack through online consultation via WA and telephone” (P5)*.

### 3. Implementing Family Nursing Care

The theme described the implementation of family nursing care to address the existing health problem. In this third theme, two sub-themes were identified: educating families on how to do self-isolation at home and family nursing care to increase family independence.

#### Educating Families on How to do Self-isolation at Home

Providing education for families about proper self-isolation was expressed by some participants as a way to reduce transmission. Inappropriate self-isolation behavior will result in family clusters. Participants argued that education about self-isolation at home was very important to stop the spread of the virus. Participant 1 stated: *“We provide education about what Covid-19 is. What should be done so that it does not spread to other family members” (P1)*.

### Family Nursing Care to Increase Family Independence

During the pandemic period, the CHNs provided online guidance to families for enabling comprehensive assessments and providing appropriate interventions to the family’s current health problem. This was expressed by Participant 2 : *“... then an assessment is carried out through anamnesis either online or by telephone; we ask about the patient’s general physical condition, then his/her ability to eat, and his/her independent ability to carry out his/her daily activities” (P2)*.

### 4. Spreading the Wings of Health Cadres by CHNs

This theme described the needs to empower the health cadres in managing Covid-19 in the community. Subthemes that comprised this theme were: helping CHNs to empower health cadres to educate the community regarding Covid-19; helping CHNs to empower health cadres to trace Covid-19 case in the community; and helping CHNs to empower health cadres to control stigma in the community.

#### Helping CHNs to Empower Health Cadres to Educate the Community Regarding Covid-19

The strategy carried out by the CHNs included the empowerment of health cadres in their working area to assist the nurses in giving education to the community. Participant 2 stated: *“Cadres should be more involved in (health) promotion.” (P2)*. In addition to providing health education for the community, health cadres were also involved in occupational health efforts. This was expressed by Participant 7: *“So, the information is conveyed to the occupational health effort post cadres. They post the information related to Covid-19 health education in their WhatsApp group. I send a message on the information, and they then share it” (P7)*.

Helping CHNs to Empower Health Cadres to Trace Covid-19 Case in the Community 

Nurses felt that cadres helped them in their role for tracing Covid-19 cases in the community. The empowerment of cadres to support the work of CHN is based on the fact that these cadres are the closest ones in the community. This was expressed by Participant 10: *“Every cadre…You see, each community unit has a cadre, so the nurses can get data about the community from them.” (P10)*. Having a health staff in the neighborhood helped the CHNs to trace the cases in the community. Each local cadre is familiar with his/her area, so that the work of the CHN can be done more efficiently.

### Helping CHN to Empower Health Cadres to Control Stigma in the Community

CHNs can empower the cadres to bridge communication between the nurses and community. This statement was expressed by participant 9: *“Cadres can become the facilitator for communication, so that the patients who are tested positive for Covid-19 do not get stigmatized in the community “ (P9)*. The presence of cadres who were also a part of the community paved the way to approach the community members. This was supported by the statement from Participant 7: *“Then, we asked the cadres to provide direct education to the community, right, because the cadres are close to the community; it is hoped that the approach will be easier and no negative perspective will rise in the community”(P7)*.

### 5. Collaboration as the Heart of Covid-19 Prevention and Management

This theme explained that collaboration as the heart of Covid-19 prevention and management in the community setting. Subthemes that comprised this theme were: collaboration with Non-Government Organizations (NGO) to train CHC health workers on 3T (Tracing, Testing, Treatment); Collaboration with NGO for health education and community-based surveillance training for people in the community; Collaboration with the local Covid-19 task force; and Optimizing of human resource allocation through collaboration.

#### Collaboration with Non-Government Organization (NGO) to Train CHC Health Workers on 3T (Tracing, Testing, Treatment)

Collaboration with NGO can assist the CHNs in responding Covid-19 in the community. Collaboration with NGO can strengthen the CHC capacity through provision of training for health workers. Participant 6 stated: *“ Pencerah Nusantara is a program to strengthen the capacity of community health center by assigning the health workers in the CHC. This program happens to be implemented by an NGO called CISDI (Center for Indonesia’s Strategic Development Initiatives), a non-profit NGO that is focused on strengthening the CHC capacity in responding to Covid at the primary health care level. This is done through training for health workers” (P6)*.

The training providing by CISDI aimed at building the capacity of the health care workers at the CHC level on implementing the 3T. Health care workers were given training from qualified sources regularly every month.

### Collaboration with NGO for Health Education and Community-based Surveillance Training for People in the Community

Collaboration with NGO helps to boost the performance of the CHC in managing Covid-19 pandemic through community empowerment. Participants revealed that the CISDI NGO, through “National Enlighten” movement, had performed community-based health education training. Participant 2 stated: *“People are provided with knowledge about information related to Covid-19, basic knowledge about Covid, risk communication, then 3T, the terms used in covid pandemic, and how to educate the public on health and other topics “ (P2)*. Apart from this, there was also community-based surveillance training to improve the performance of CHC in tracing. Participant 2 expressed: *“Community-based surveillance is about how to empower the community, in this case volunteers, to assist the CHC in conducting tracing, which means the possibility to identify more suspects in the community is higher” (P2)*.

### Collaboration with the Local Covid-19 Task Force

CHNs need to collaborate with other parties to manage Covid-19. The involvement of the local communities through the local Covid-19 task force in the area is required. Participant 10 stated: *“CHNs collaborates with Covid task force in each area when providing information about Covid” (P10)*. Participants also mentioned that the local Covid-19 task force helped the nurses in monitoring compliance during self-isolation in the community. Participants 7 stated: *“ Together with the task force, you see, in most of locations that we will be assisted by the Local Neighborhood Leader if there are patients who are in self-isolation, right, help to monitor (P7)”*. Apart from this, Participant 6 stated: *“We collaborate with local officials who can play a role and live nearby the patient’s house, or their closest family. For example, there is a patient who is currently in isolation; what about his or her logistical needs, so that it requires collaboration across sectors, right? ”(P6)*.

Another local element involved in Covid-19 management is law enforcement. Participants stated that in enforcing the discipline of health protocol implementation in the community, the support from law enforcement agents such as the armed force/police was needed to optimize the compliance. Participant 4 stated: *“ The shop also followed the rule to only open until 8:00 p.m. It is also important to coordinate with the local law enforcement officers from the police or the armed force.” (P4)*.

### Optimizing of Human Resource Allocation Through Collaboration

This sub-theme was raised with reference to the limited availability of human resources for implementing CHN services at the CHC level. Participants stated that they always optimized the use of the available resources to run the program. This was expressed by Participant 3: *“But we try to use and optimize the existing human resources so that all activities can be run smoothly, you see. There is no reason for activities to be late due to lack of human resources(P3)*. Collaboration with local community was also performed to overcome the obstacles or difficulties they encountered. Participant 4 stated: *“ Through multi-sector approach, yes, so we collaborate, even though there are obstacles and difficulties, we look for solutions together” (P6)*.

## DISCUSSION

This qualitative study aimed to explore the roles of CHNs in the Covid-19 management in Indonesia. The results showed five main themes identified in the study, namely providing comprehensive services by CHNs; utilizing technology to bridge information needs; implementing family nursing care; spreading the wings of health cadres by CHNs; and collaborating as the heart of Covid-19 prevention and management. 

This finding is in the same line with other studies that reported the community nurses have played a key role in the integration of health and social services during the Covid-19 pandemic. ^20 , 21^ Nursing care management by adapting to the pandemic conditions can help the CHN to minimize the risk of exposure to Covid-19 in the community. Apart from this, the nurse also plays an additional role in screening potential cases, recognizing the patient’s need for self-isolation, and monitoring the cases. ^22^ CHN plays a vital role in Covid-19 pandemic management in the scope of primary health care services because they provided nursing care for individuals patients, families, and general community. ^13^ Active involvement of CHNs in the prevention and treatment of Covid-19 indicate its importance in responding to Covid-19 case.

In the current study, providing comprehensive services by CHNs is needed to control and prevent the spread of Covid-19 at the community setting. Providing comprehensive services by CHNs was also identified in CHC in Australia, indicating that the availability of services for the patients in the vulnerable group who have a higher risk of infection and require ongoing support to prevent increased morbidity is crucial. ^23^ Other studies also revealed that CHNs could also be involved in case tracing to prevent further transmission in the community. ^20 , 24^ Another finding was that the CHN can optimize the acceptability of Covid-19 vaccination, which is a very important step in preventing the spread of Covid-19. ^25^ Therefore, this emphasizes the important role of CHNs in improving health and reducing health inequality at community. 

Utilization of technology to deal with the information needs was identified in this study as one of the CHN interventions to provide nursing care services. The use of social media and teleconsultation in disseminating knowledge to the public is used as a good communication channel during the pandemic. Other qualitative studies also reported that nurses utilize digital media to spread awareness and combat misleading belief about Covid-19. ^26^ Teleconsultation can also be used to discuss health problems faced by nurse-clients. ^27^ In addition, online patient monitoring by the CHN through periodic monitoring of patients in self-isolation is also an important part of the Covid-19 management. Approaches using the digital technology can support monitoring of patients undergoing self-isolation. ^28 , 29^ We strongly recommend that dissemination of information, education and communication related to Covid-19 should be done massively and comprehensively. 

This study also found that there was a need for educating families to be able to overcome their health problems. Family nursing care can help families recover by following a range of family health tasks. The family nursing care is very much needed to increase the independence of the family, so that the family health status can be optimized. In this regard, a study found that family nursing care is important since it to helps to promote the optimal family functioning and protect the health of family members. ^30^ The family plays a crucial role in implementation of health care practices, including preventing health problems or caring for sick family members. ^31^ During the period of self-isolation, the importance of educating family is emphasized as it will ensure the compliance with self-isolation. ^31 , 32^

According to the findings, empowerment of health cadres is basically needed as a strategy to change the people’s perspective, encourage health behavior, and minimize stigma in the community. Based on this study, spreading the wings of health cadres is important in Covid-19 management in the community. Health cadres can assist in providing support related to Covid-19 under the CHN implementation. This finding is in line with the efforts to handle Covid-19 in Thailand which involved village health cadres to support surveillance in the community. Health cadres can also assist health professionals in carrying out proactive screening to detect the early spread of the virus in the community. ^33^ Furthermore, health cadres can also help to overcome social stigma in the community. ^34^ In addition, the majority of health cadres already have good knowledge about Covid-19 and have ample opportunities to provide Covid-19 education to the community. ^35^

The results of the present study showed that collaboration is the heart of Covid-19 prevention and management. Collaboration is also established between the CHNs and non-government organization (NGO) to help control the Covid-19 case in the community. This finding in congruent with other studies that reported collaboration with NGO would help the CHNs in performing their job in managing Covid-19 as well as supporting the public health service system and community involvement to carry out various prevention and control activities. ^36 , 37^ In addition, another study found that, the past public health emergency of Ebola in West Africa highlighted the need for community engagement strategies to carry out various prevention and control activities. ^36^ Therefore, collaboration between programs and related sectors to implement the CHN interventions requires cooperation, support, and integration from all parties to be able to meet the increasingly complex health needs. ^38^

The main strength of this study was that it presented the roles of CHNs through the implementation of various intervention strategies in Covid-19 management in Bandung City, West Java, Indonesia. The strategy of CHN through health education, empowerment, group processes, and advocacy can be adapted to the conditions of the Covid-19 pandemic and optimize Covid-19 management at the community level. The CHNs play a vital role in the prevention and control of Covid-19 by providing care to Covid-19 patients, their families, and the community. Therefore, this present study can be used in policy-making and planning for Covid-19 management. 

The findings of this study can be used as a lesson learned regarding the CHN interventions in dealing with Covid-19 pandemic. The results of this qualitative study may not represent a general picture of all community health services. Thus, the findings are limited to a particular region and may not be generalizable for the global population.

## CONCLUSION

Active engagement of CHNs in the primary health care system plays essential roles in preventing and controlling Covid-19. CHN has essential tasks in managing Covid-19 through providing comprehensive services by CHNs, utilization of technology, family nursing care, community empowerment, and multi-program and multi-sector collaborations. These findings can be used as inputs for policy makers when developing strategies to optimize CHN implementation amidst Covid-19 and possible future global pandemic challenges. Further research is needed to integrate the CHNs as the integral part of Covid-19 management as well as prevention of other health problems in the community.

## ACKNOWLEDGEMENT

The present article is the result of Master of Nursing Thesis approved by Universitas Padjadjaran, with Basic Research Excellence Grant from Ministry of Education, Cultural, Research, and Technology No. 1207/UN6.3.1/PT.00/2021. We would like to thank Bandung City Health Office, Garuda Community Health Center, Kopo Community Health Center, and Griya Antapani Community Health Center for their contributions. We are also grateful to Laili Rahayuwati, Dr.PH; Iqbal Pramukti, Ph.D; and Mamat Lukman, M.Si who provided inputs for this study. 

**Conflict of Interest:** None is declared. 
